# Mutational status of *TP53* defines the efficacy of Wee1 inhibitor AZD1775 in *KRAS*-mutant non-small cell lung cancer

**DOI:** 10.18632/oncotarget.18728

**Published:** 2017-06-28

**Authors:** Bo Mi Ku, Yeon-Hee Bae, Jiae Koh, Jong-Mu Sun, Se-Hoon Lee, Jin Seok Ahn, Keunchil Park, Myung-Ju Ahn

**Affiliations:** ^1^ Samsung Biomedical Research Institute, Department of Medicine, Samsung Medical Center, Sungkyunkwan University School of Medicine, Seoul, Korea; ^2^ Division of Hematology-Oncology, Department of Medicine, Samsung Medical Center, Sungkyunkwan University School of Medicine, Seoul, Korea

**Keywords:** NSCLC, Wee1, AZD1775, KRAS, TP53

## Abstract

*KRAS* is frequently mutated in non-small cell lung cancer (NSCLC). However, direct targeting of KRAS has proven to be challenging, and inhibition of KRAS effectors has resulted in limited clinical efficacy. Wee1 kinase is an important regulator of the G2 checkpoint and is overexpressed in various cancers. Inhibition of Wee1 exerts anticancer effects as a monotherapy or in combination with DNA-damaging agents when cancer cells harbor *TP53* mutations. However, its role in *KRAS*-mutant NSCLC, especially as a single agent, has not been explored. Here, we investigate the anticancer potential of Wee1 inhibitor AZD1775 as a monotherapy and uncover a possible cellular context underlying sensitivity to AZD1775. Our data show that treatment with AZD1775 significantly inhibited cell survival, growth, and proliferation of *TP53*-mutant (*TP53*^MUT^) compared to *TP53* wild-type (*TP53*^WT^) in *KRAS*-mutant (*KRAS*^MUT^) NSCLC cells. In *KRAS*^MUT^/*TP53*^MUT^ cells, AZD1775 treatment led to DNA damage, a decrease of survival signaling, and cell death by apoptosis. Interestingly, cell death through apoptosis was found to be heavily dependent on specific cellular genetic context, rather than inhibition of Wee1 kinase activity alone. In addition, AZD1775 treatment was well tolerated and displayed single-agent efficacy in a mouse xenograft model. This study provides rationale for inhibiting Wee1 using AZD1775 as a potential anticancer therapy against the *TP53*^MUT^ subgroup of *KRAS*^MUT^ NSCLC.

## INTRODUCTION

*KRAS* is the most frequently mutated oncogene in non-small cell lung cancer (NSCLC), and activating KRAS mutations predict poor outcome in response to conventional treatment regimens [1–3]. Although targeting KRAS effector pathways has been suggested as a therapeutic alternative, there are no approved targeted therapies for the treatment of *KRAS*-mutant (*KRAS*^MUT^) cancers. Thus, *KRAS*^MUT^ NSCLC remains a clinical challenge. Oncogenic KRAS expression is frequently associated with increased DNA damage through replicative stress [3, 4]. In response to DNA damage or replication stress, *KRAS*-driven cancer cells activate G2 checkpoint kinases, such as ATR, Chk1, and Wee1, which might promote the survival of these cells [3, 5]. Therefore, G2 checkpoint kinase inhibitors have been suggested as therapeutics for targeting *KRAS*-driven cancers [6]. Recently, the Wee1 inhibitor AZD1775 was identified as a potential agent for targeting mutant KRAS-expressing cancers [3, 7].

Wee1 is a protein kinase and inhibitory regulator of the G2/M cell cycle checkpoint. During a normal G2/M transition, Plk1 phosphorylates Wee1, which targets Wee1 for degradation via the ubiquitin ligase complex. In the presence of DNA damage, ATM/ATR pathways negatively regulate Plk1 and stabilize Wee1 to cope with DNA damage [8, 9]. In response to DNA damage, Wee1 inactivates CDK1 through phosphorylation at Tyr15, thereby preventing cells from proceeding through mitosis by maintaining G2 arrest. This G2 arrest gives rise to a survival advantage in tumor cells by allowing them time to repair their damaged DNA [8]. In addition, Wee1 stabilizes DNA replication forks during the S phase of the cell cycle via a CDK2 mechanism [8, 10]. Inhibition of Wee1 has been expected to abrogate the G2/M checkpoint, forcing tumor cells with DNA damage to enter into unscheduled mitosis to undergo cell death [11–13]. Wee1 overexpression has been observed in several cancers, and high Wee1 expression has also been shown to correlate with tumor progression [8, 14–16]. In addition, high expression of Wee1 has been observed in response to elevated replication stress [8].

Preclinical efficacy of the Wee1 inhibitor AZD1775, also known as MK1775, was assessed in several cancers on the basis of the concept of synthetic lethality. In these studies, AZD1775 abrogated the G2 checkpoint arrest and selectively sensitized p53-deficient cells to various DNA-damaging agents [17–20]. Furthermore, a recent clinical phase II study reported that AZD1775 enhances carboplatin efficacy in *TP53*-mutant (*TP53*^MUT^) ovarian cancer [21]. Together, these studies suggest that treatment with AZD1775 in combination with DNA-damaging agents is particularly effective against *TP53*^MUT^ cancers. *TP53* is the most commonly mutated gene in human cancers and is a significant player in G1/S checkpoint regulation. DNA damaged cells that have mutations in *TP53* rely extensively on the G2 checkpoint for survival. Thus, abrogation of the G2 checkpoint might preferentially sensitize *TP53*-mutant cancers to DNA-damaging agents [5]. However, other studies have shown that inhibition of Wee1 is also effective in *TP53* wild-type (*TP53*^WT^) contexts, implying that the mechanism of action is independent of *TP53* status [8, 22, 23]. In addition, a recent phase I trial showed that some patients obtained benefits from AZD1775 monotherapy for a prolonged period of time, regardless of *TP53* mutational status [24]. Taken together, these findings indicate that it is unlikely that *TP53* status alone can fully predict the efficacy of Wee1 inhibitors in cancer. Nevertheless, certain *TP53* mutations might act as one of several factors that contribute to an increase in the efficacy of Wee1 inhibitors.

Although the antitumor effects of AZD1775 have been usually tested in combination with chemotherapy, several studies have shown that AZD1775 exerts potent cytotoxic effects as a single agent in sarcoma, glioblastoma, neuroblastoma, and HNSCC [19, 22, 25, 26]. However, the single-agent efficacy of AZD1775 in *KRAS*^MUT^ NSCLC remains largely unknown. Therefore, we investigated the efficacy of AZD1775 in the context of *TP53* status in *KRAS*^MUT^ NSCLC. Results obtained from this study provide evidence that AZD1775 can be effective in treating a subset of NSCLC harboring concomitant *KRAS* and *TP53* mutations.

## RESULTS

### Cytotoxic effects of AZD1775 are dependent on *TP53* status in *KRAS*-mutant NSCLC cells

**Figure 1:**
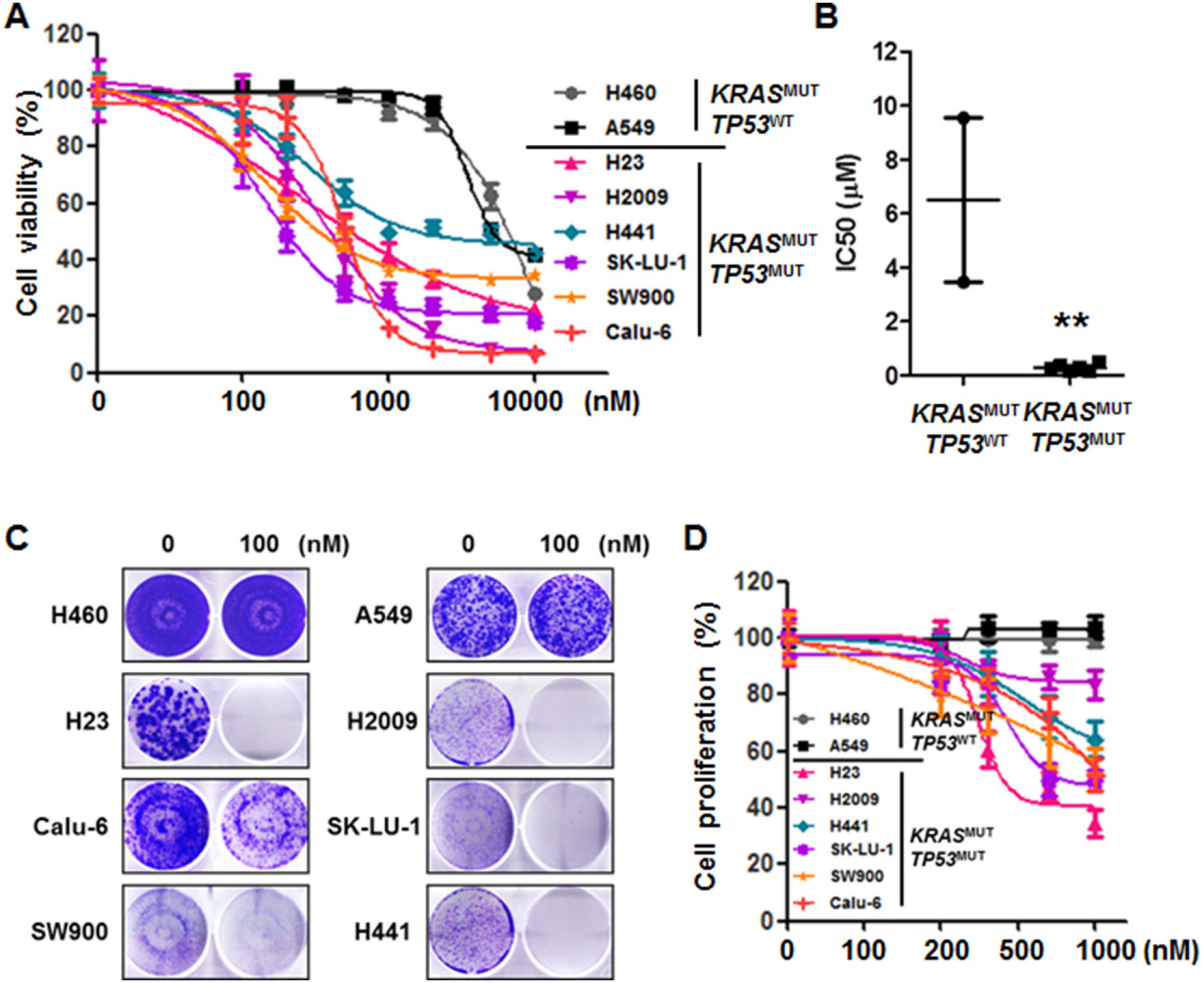
Mutational status of TP53 determines the effectiveness of AZD1775 in KRAS^MUT^ lung cancer **(A)** KRAS^MUT^ lung cancer cell lines were treated with the indicated concentrations of AZD1775 for 72 h. Cell viability was determined using a CCK-8 assay. Data are presented as mean ± SE (n=6). **(B)** KRAS-mutant cell lines were divided into TP53^WT^ and TP53^MUT^ groups. The dots represent the IC_50_ of each cell line, and the bars indicate the means. **, *P*<0.01. **(C)** Cells were treated with 100 nM AZD1775 for 14 days and then stained with crystal violet. A representative plate is shown. **(D)** After 48 h of treatment, cell proliferation was determined by BrdU incorporation. Data are presented as mean ± SE (n=8). MUT, mutant; WT, wild-type.

To investigate the cellular response to AZD1775 and explore the contributions of *KRAS* genomic status to its effects, we used *KRAS*^MUT^ NSCLC cell lines with previously defined *TP53* and *STK11* status. For evaluation of the cytotoxic effects of AZD1775, eight *KRAS*^MUT^ NSCLC cell lines were treated for 72 h. As shown in Figure 1A, the *TP53*^MUT^ cell lines (H23, H2009, H441, SK-LU-1, SW900, and Calu-6) were significantly more sensitive to AZD1775 than the *TP53*^WT^ cell lines (H460 and A549). Comparing the mean IC_50_ values of the two groups revealed that the *TP53*^MUT^ group had a much lower mean IC_50_ (0.277±0.054) than that of the *TP53*^WT^ group (6.498±3.043) (Figure 1B). When given as a long-term treatment, AZD1775 inhibited colony formation only in the *TP53*^MUT^ group, (Figure 1C). In addition, cell proliferation was significantly reduced in the *TP53*^MUT^ group, but not in the *TP53*^WT^ group (Figure 1D). However, there was no correlation between *STK11* mutation status and cytotoxic effects of AZD1775. These results suggest that AZD1775 is effective as a single agent in a subtype of NSCSL carrying concomitant *KRAS* and *TP53* mutations, regardless of the coexistence of *STK11* mutation.

### Cytotoxic effects of AZD1775 are kinase activity independent

**Figure 2:**
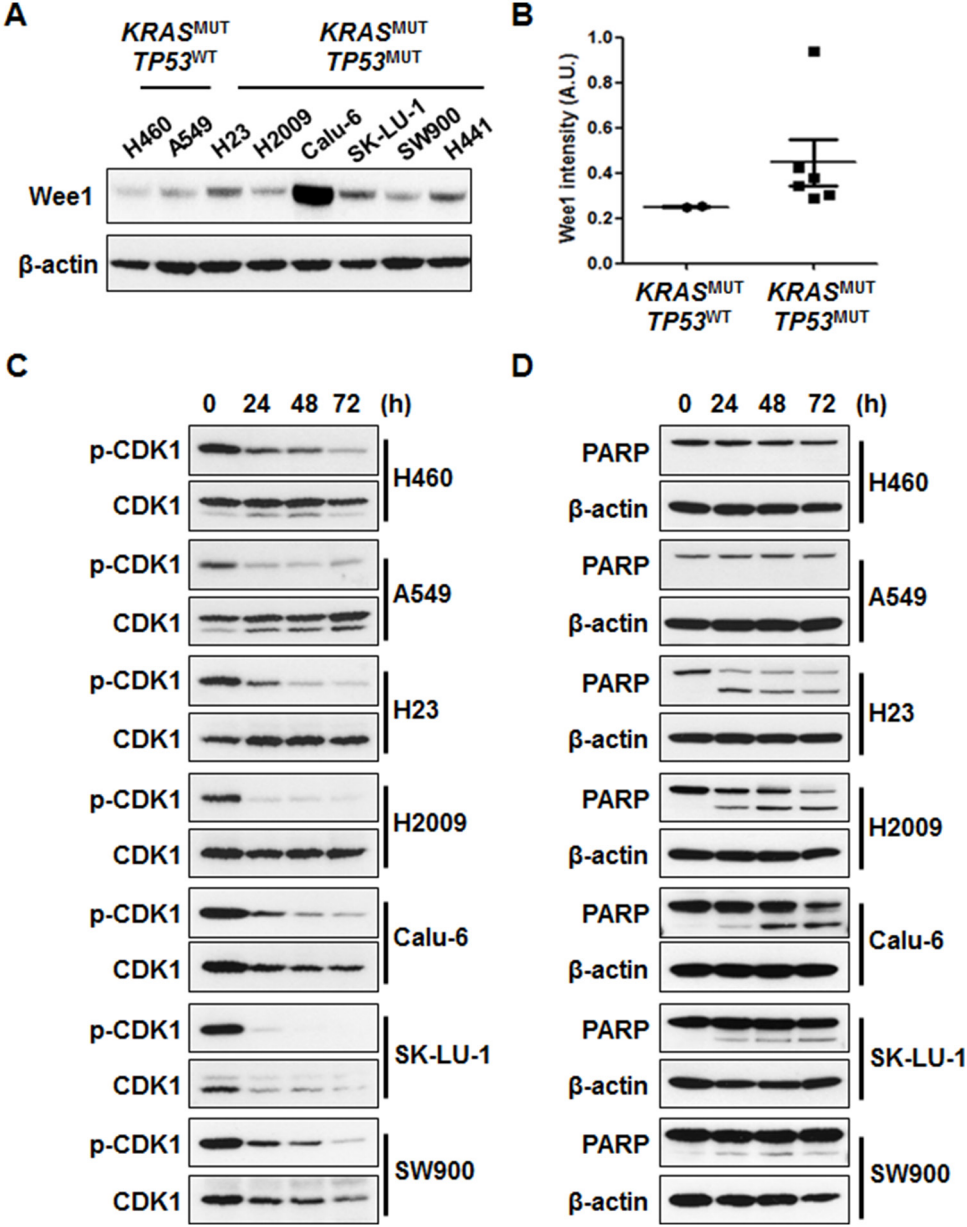
AZD1775 induces cell death regardless of p-CDK1 inhibition **(A)** Cell lysates were subjected to immunoblot analysis to detect Wee1 expression. **(B)** The graphs represent densitometric quantification of Wee1 immunoblot bands. **(C** and **D)** Cells were treated with 500 nM AZD1775 for the indicated times. Phosphorylation of CDK1 (C) and PARP cleavage (D) was assessed using Western blots. MUT, mutant; WT, wild-type.

**Figure 3:**
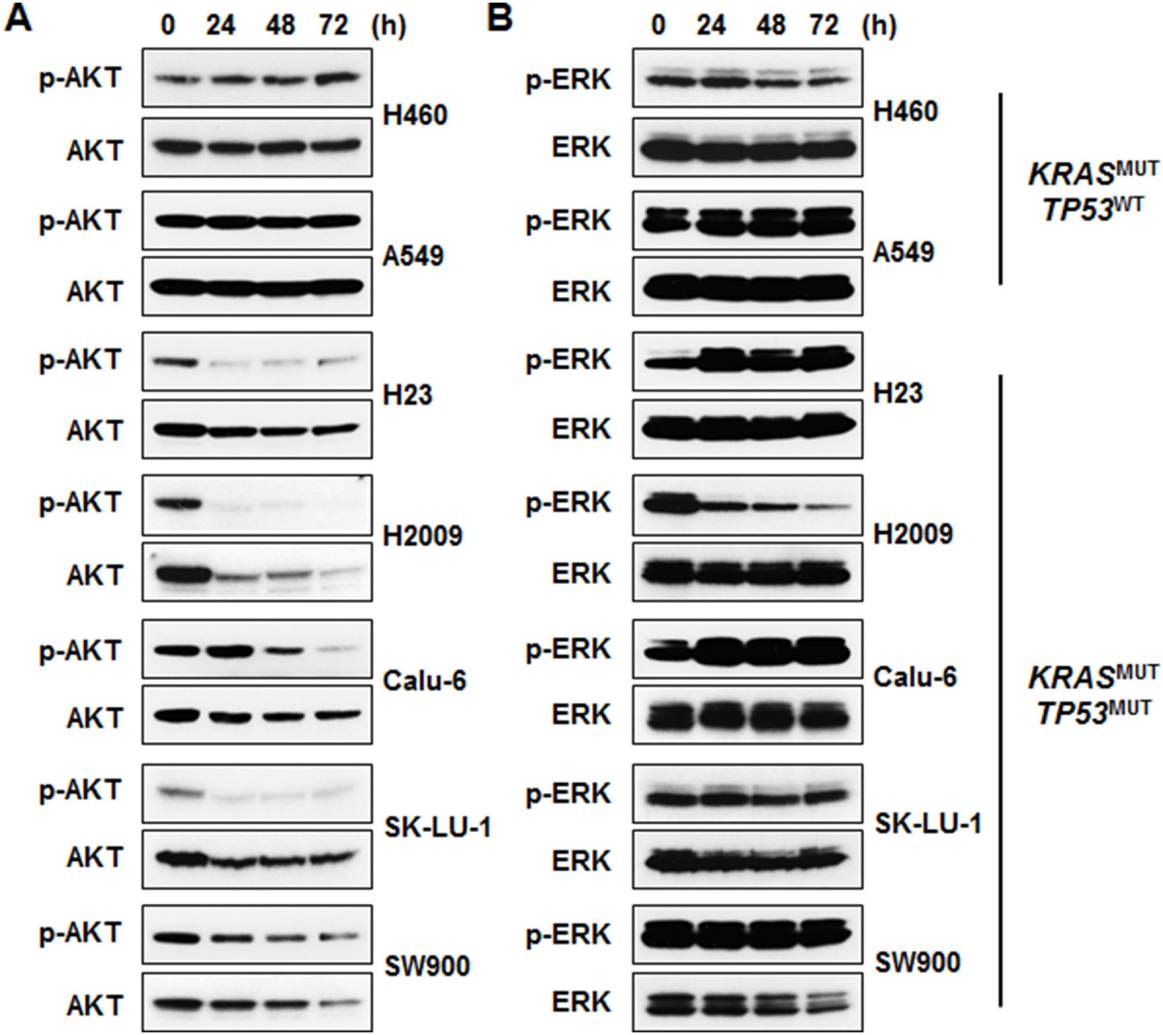
AKT signaling is involved in the sensitivity of KRAS^MUT^/TP53^MUT^ lung cancer to AZD1775 Cells were treated with 500 nM AZD1775 for the indicated times. **(A)** Phosphorylation of AKT was determined by immunoblotting. **(B)** Phosphorylation of ERK was determined by immunoblotting. MUT, mutant; WT, wild-type.

To elucidate the molecular mechanisms of AZD1775, we conducted Western blot analysis. Although Wee1 expression level was slightly higher in *TP53*^MUT^ cell lines, this difference was not statistically significant (Figures 2A and 2B). The ability of AZD1775 to inhibit Wee1 kinase activity was assessed using p-CDK1 (Tyr15) as a surrogate. After AZD1775 treatment, all *KRAS*^MUT^ NSCLC cell lines showed a dramatic decrease in p-CDK1 level irrespective of *TP53* mutation status (Figure 2C). Time-course analysis of p-CDK1 protein expression level revealed that Wee1 inhibition by AZD1775 maintained this downregulation for 72 h (Figure 2C). However, p-CDK1 level had no correlation with cell viability when AZD1775 was used as a single agent: PARP cleavage was only detected in *TP53*^MUT^ cells, but not in *TP53*^WT^ cells (Figure 2D). In signal pathway analysis of cell survival using p-AKT and p-ERK, AZD1775 significantly reduced p-AKT level in *TP53*^MUT^ cells (Figure 3A). In contrast, except in H2009 cells, p-ERK level was not significantly changed by AZD1775 treatment in any of the tested cell lines (Figure 3B). These results indicate that the kinase activity of Wee1 might not be involved in the apoptotic response to AZD1775 as a single agent in *KRAS*^MUT^ NSCLC.

### DNA damage underlies AZD1775-induced cytotoxicity

**Figure 4:**
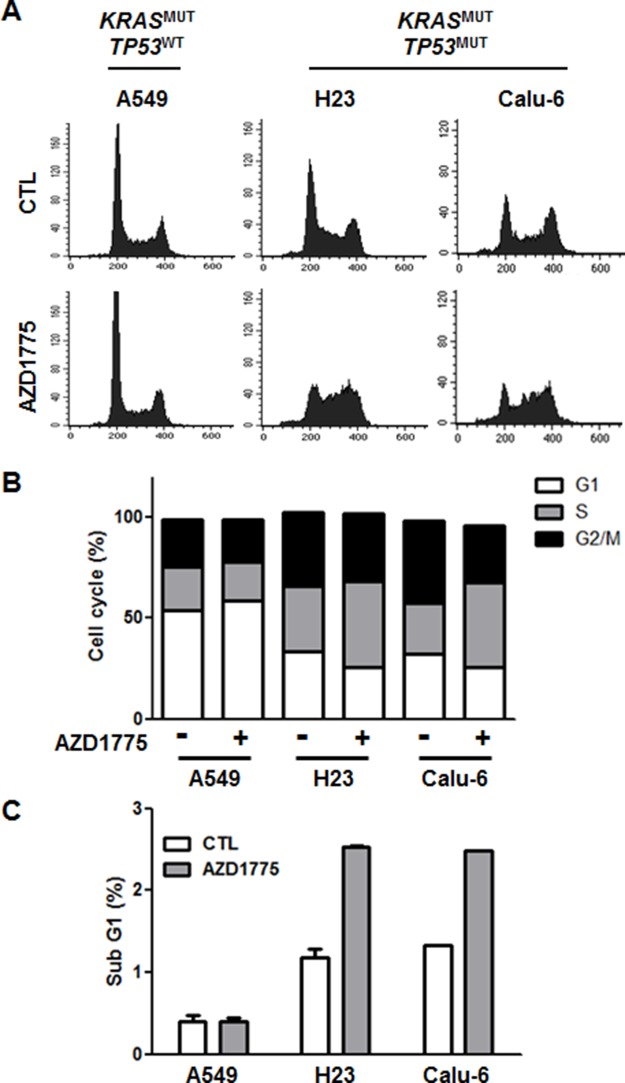
AZD1775 has no effect on cell cycle A549, H23, and Calu-6 cells were treated with 500 nM AZD1775. After 24 h treatment, cell cycle analysis was performed using PI staining followed by flow cytometry. **(A)** Histogram representing the distribution of cell cycle. **(B)** The percentage distribution of cells in the G0/G1, S, and G2/M phases are shown. Data represent mean ± SEM (n = 3). **(C)** The bars represent the percentage of cells in the sub-G1 fraction in each cell lines. MUT, mutant; WT, wild-type.

**Figure 5:**
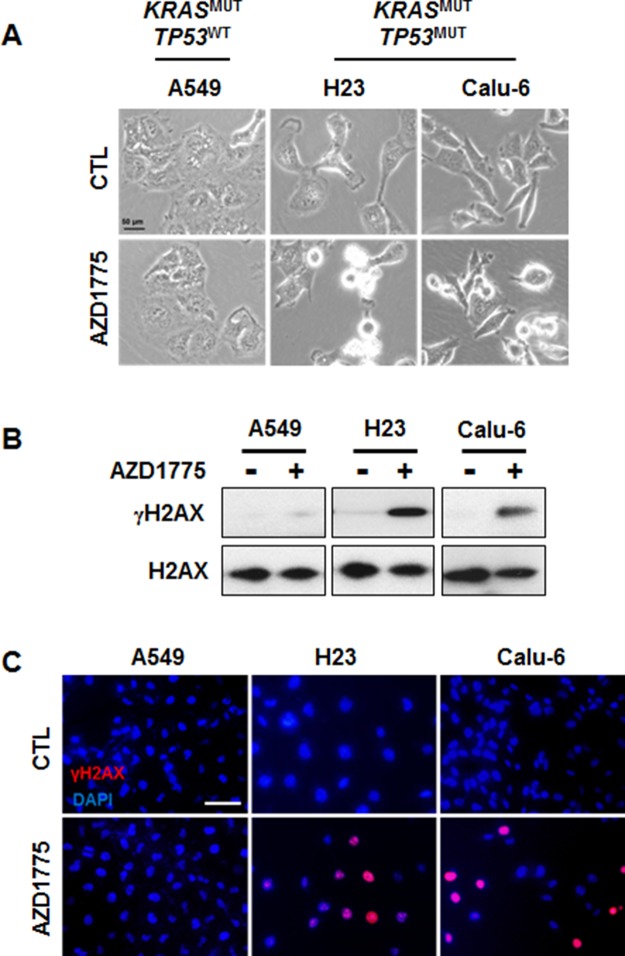
AZD1775 treatment causes DNA damage in KRAS^MUT^/TP53^MUT^ lung cancer A549, H23, and Calu-6 cells were treated with 500 nM AZD1775 for 24 h. **(A)** Phase-contrast image of cells. **(B)** Expression levels of γH2AX and H2AX were analyzed by Western blotting. **(C)** Cells were stained for γH2AX (Alexa Fluor 594, red), and nuclei were counterstained with DAPI. Scale bar, 50 μm. MUT, mutant; WT, wild-type.

Next, we choose three representative cell lines, A549, H23, and Calu-6, to further investigate the molecular mechanisms of AZD1775. Because Wee1 is required for the regulation of both the S and G2 phases of the cell cycle, inhibition of Wee1 is expected to lead to both S phase defects (DNA double-strand breaks) and G2/M defects (premature mitosis) [22]. To test whether either or both of these effects is necessary for AZD1775-induced cytotoxicity, we analyzed cell cycle and apoptosis after treatment with this compound. While no cell cycle change was observed in the *TP53*^WT^ A549 cell line, the *TP53*^MUT^ H23 and Calu-6 cell lines showed a slight increase of in the percentage of cells in the S phase after 24 h of treatment (Figures 4A and 4B). Accordingly, the sub G1 population was significantly increased by AZD1775 treatment only in *TP53*^MUT^ cells (Figure 4C). Consistent with apoptotic cell death (Figure 5A), AZD1775 treatment also led to an increase in the S139 phosphorylation of H2AX (γH2AX) (Figures 5B and 5C). Moreover, AZD1775 induced pan-nuclear γH2AX staining without visible foci, which is consistent with previous reports (Figure 5C) [12, 27]. Increased γH2AX level indicates that AZD1775 might cause DNA damage through replication stress.

### AZD1775 monotherapy has antitumor activity *in vivo*

**Figure 6:**
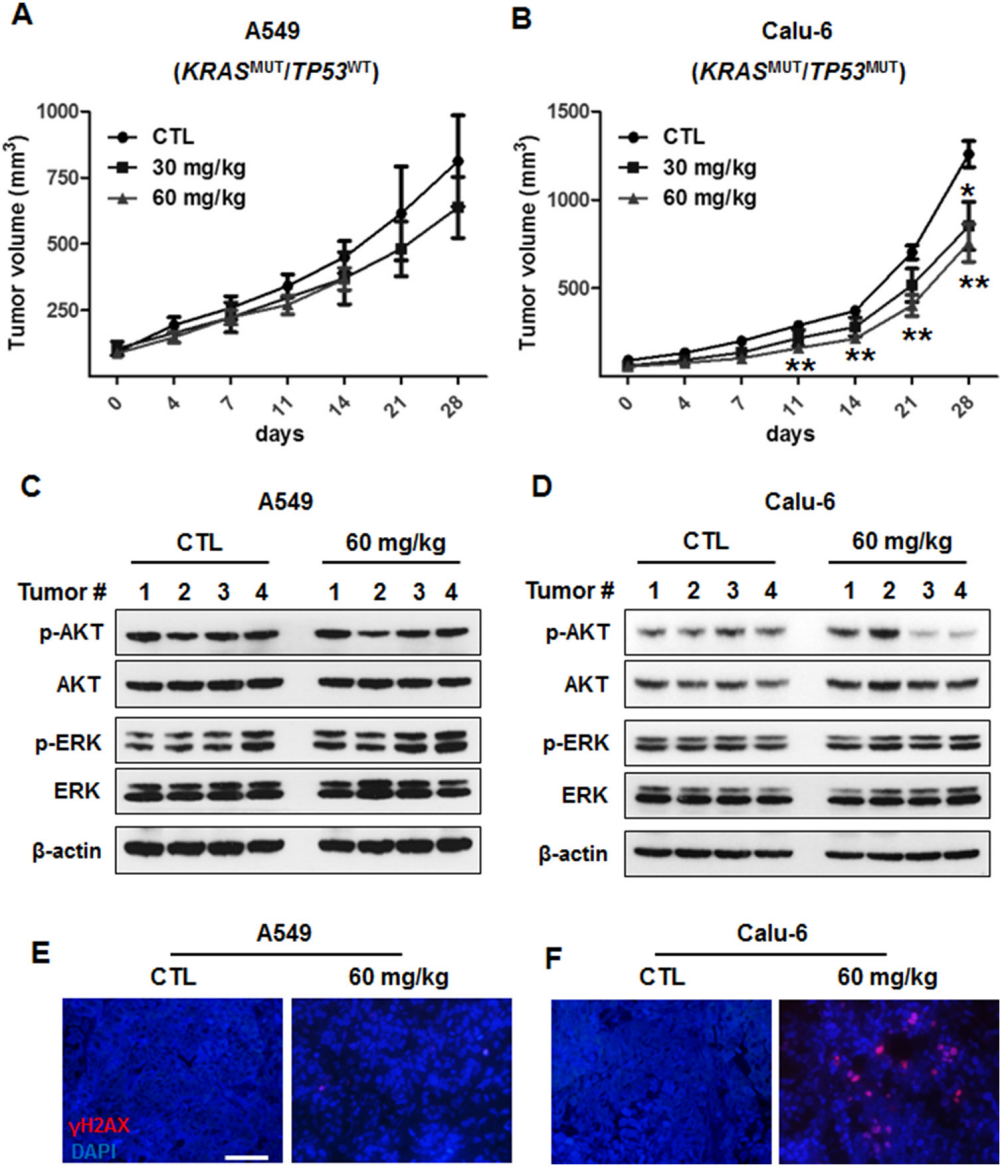
*In vivo* antitumor efficacy of AZD1775 in xenograft models of KRAS^MUT^/TP53^WT^ and KRAS^MUT^/TP53^MUT^ lung cancer A549 (KRAS^MUT^/TP53^WT^) and Calu-6 (KRAS^MUT^/TP53^MUT^) cells were subcutaneously injected into the flanks of Balb/c nude mice. **(A and B)** A549 (A) and Calu-6 (B) xenograft-bearing mice were treated with either vehicle (once a day) or AZD1775 (30 mg/kg;once a day and 60 mg/kg; twice a day) by oral gavage. Tumor volumes were measured twice weekly, and data are presented as mean ± SE (n=5-7). *, *P* < 0.05; **, *P* < 0.01. **(C and D)** A549 (C) and Calu-6 (D) tumor lysates were immunoblotted with the indicated antibodies, and β-actin was used as a loading control. **(E and F)** A549 (E) and Calu-6 (F) tumor sections were stained with γH2AX (Alexa Fluor 594, red). Nuclei were counterstained with DAPI. Scale bar, 50 μm. MUT, mutant; WT, wild-type.

To evaluate the efficacy of AZD1775 in *KRAS*^MUT^ NSCLC *in vivo*, we generated tumor xenografts derived from A549 (*TP53*^WT^) and Calu-6 (*TP53*^MUT^) cells. Mice bearing tumor xenografts were treated with a vehicle control (once daily) or AZD1775 (30 mg/kg, once daily or 60 mg/kg, twice daily). This dosing strategy was tolerable, and no body weight loss or other signs of toxicity were observed. In line with *in vitro* data, AZD1775 significantly decreased tumor growth of Calu-6 xenografts after 11 days of treatment, but had no effects on A549 xenografts (Figures 6A and 6B). Along with inhibiting tumor growth, AZD1775 treatment reduced the level of p-AKT in Calu-6 xenografts, but had no effect on p-ERK activation in either xenograft model. In addition, AZD1775-treated Calu-6 tumors showed significantly greater levels of replication stress, as measured by γH2AX pan-nuclear staining (Figures 6E and 6F). Taken together, these results suggest that AZD1775 is a potential therapeutic option for NSCLC patients carrying concomitant *KRAS* and *TP53* mutations.

## DISCUSSION

Replication stress is a common feature of oncogene-driven NSCLC, which could sensitize cells to checkpoint kinase inhibition by enhancing S phase damage [5, 6, 13]. Activating mutations in *KRAS* are the most common oncogenic driver in NSCLC and account for 20-30% of NSCLC cases. Recently, it has been reported that *KRAS*^MUT^ cell lines display intrinsic genotoxic stress, and that checkpoint kinase inhibitions have synergistic cytotoxic effects in these cells [28]. Also, studies have suggested that *KRAS*^MUT^ cell lines might adapt to oncogenic stress by simultaneous inactivation of *TP53* [28]. Recently, Skoulidis *et al*. demonstrated that co-occurring genetic alterations in *STK11*, *TP53*, and *CDKN2A/B* define three major subgroups of *KRAS*^MUT^ lung adenocarcinoma with distinct biology and therapeutic vulnerabilities [29]. In drug sensitivity analysis, *KRAS*^MUT^/*STK11*^MUT^ cells showed increased vulnerability to HSP90-inhibitor therapy [29]. Other reports have shown that *KRAS*^MUT^/*TP53*^MUT^ and *KRAS*^MUT^/*STK11*^MUT^ lung cancers presented markedly different responses to docetaxel monotherapy in a mouse model [30]. These results suggest that this classification scheme could be useful in guiding treatment strategies.

Initially, it was believed that sensitivity to Wee1 inhibition was associated with an aberrant G1 checkpoint caused by a mutation in *TP53* [8, 23, 31]. However, many studies have subsequently shown that inhibition of Wee1 is also effective in *TP53*^WT^ contexts, suggesting the effects of this inhibition are independent of *TP53* status. A Wee1 inhibitor as a monotherapy has also been found to be effective in the absence of DNA-damaging agents. In a clinical study, AZD1775 as a single agent was well tolerated in patients, with a favorable adverse effect profile [24]. Although Wee1 inhibitors are currently being tested in several clinical trials, predictive biomarkers for sensitivity to Wee1 inhibitors have not been fully elucidated [8]. It is important to note that *TP53* is the only biomarker currently being employed in clinical trials assessing AZD1775 therapy [21].

Based on these results, we investigated whether Wee1 inhibition might have a different therapeutic efficacy in *KRAS*^MUT^ NSCLC depending on *TP53* status. In this study, we identified that AZD1775 as a single agent is significantly more cytotoxic to *KRAS*^MUT^/*TP53*^MUT^ than to *KRAS*^MUT^/*TP53*^WT^ NSCLC cell lines. The cytotoxic effects of AZD1775 were not dependent on inhibition of Wee1 kinase activity alone. The anticancer effects of this Wee1 inhibitor might be related to the genomic instability of the cancer cells, which results in sub-lethal DNA damage, rather than the G2 checkpoint kinase activity. Despite the profound growth inhibitory effects observed *in vitro*, the effects of AZD1775 monotherapy on tumor inhibition *in vivo* were moderate in this study. Since Calu-6 cells are known to easily establish tumor xenografts in nude mice, we choose this cell line for *in vivo* study. However, Calu-6 cells showed more rapid formation of colonies in the colony formation assay and persistent AKT activation after AZD1775 treatment compared with other *KRAS*^MUT^/*TP53*^MUT^ NSCLC cells. These characteristics of Calu-6 cells could explain the moderate *in vivo* effects of AZD1775 seen in this study. The induction of γH2AX in *KRAS*^MUT^/*TP53*^MUT^ NSCLC cells by AZD1775 suggests that DNA damage by replication stress is the dominant mechanism underlying the effectiveness of AZD1775. In concordance with our results, several studies have reported that Wee1 inhibition induces replication stress [10, 13, 25, 32, 33]. AZD1775 has been shown to result in an increase in γH2AX level in breast carcinoma cells, with concomitant accumulation of cells in the S phase [34]. Similar results have also been obtained using siRNA against Wee1 in human osteosarcoma cells [35]. AZD1775 has been shown to cause excess origin firing, caused by an increase in active CDK1, which leads to nucleotide exhaustion and double-strand DNA breaks [36]. Preclinical and clinical research has demonstrated that AZD1775, acting as a single agent, is associated with the role of Wee1 in the stabilization of replication forks and homologous recombination repair [8, 24, 31]. Especially, in a phase I study of AZD1775 as a single agent, Do *et al*. reported one NSCLC patient with a *KRAS*^MUT^/*TP53*^WT^ status who showed concurrent increases in p-HH3 and γH2AX levels in the post-treatment tumor biopsy compared with baseline levels [24].

A previous study indicated that lack of Wee1 expression correlated inversely with prognosis in NSCLC [17]. *TP53*^MUT^ NSCLC cell lines appeared to proceed more frequently into mitosis in the presence of AZD1775, either as a monotherapy or combined with irradiation, compared with untreated cells, indicating abrogation of G2 arrest. Furthermore, AZD1775 enhanced the antitumor efficacy of irradiation in a NSCLC xenograft model [20]. In addition, various studies reported the application of Wee1 inhibitor as monotherapy or in combination with chemotherapy or radiation therapy [8, 10, 11, 25]. Thus, combining AZD1775 with other chemotherapeutic agents or targeted therapies may be helpful for the treatment of patients with *TP53*^MUT^ NSCLC.

In conclusion, we demonstrated that AZD1775 is significantly more cytotoxic to *TP53*^MUT^ than to *TP53*^WT^ NSCLC both *in vitro* and *in vivo* in a *KRAS*^MUT^ context. The underlying mechanism of this cytotoxicity appears to be related to DNA damage and inhibition of AKT signaling. Our results show that the effect of Wee1 inhibition is not limited to *TP53* mutation, but is heavily dependent on specific cellular context. These preclinical data provide compelling evidence that a personalized approach to the treatment of *KRAS*^MUT^ NSCLC with a Wee1 kinase inhibitor in *TP53*-mutated cells might be feasible. Based on these results, further clinical study of AZD1775 in NSCLC patients with *KRAS*^MUT^/*TP53*^MUT^ is warranted.

## MATERIALS AND METHODS

### Cell cultures

The human NSCLC cell lines H460 (KRAS^Q61H^/TP53^WT^/STK11^Q37^*), A549 (KRAS^G12S^/TP53^WT^/STK11^Q37^*), H23 (KRAS^G12C^/TP53^M246I^/STK11^W332^*), H2009 (KRAS^G12A^/TP53^R273L^/STK11^WT^), H441 (KRAS^G12V^/TP53^R158L^/STK11^WT^), SK-LU-1 (KRAS^G12D^/TP53^H193R^/STK11^WT^), SW900 (KRAS^G12V^/TP53^Q167^*/STK11^WT^), and Calu-6 (KRAS^Q61K^/TP53^R196^*/STK11^WT^) were obtained from the American Type Culture Collection (ATCC). Cells were cultured in RIMI1640 containing 10% FBS at 37°C in a humidified atmosphere containing 5% CO_2_. These cell lines were authenticated by short tandem repeat profiling at Samsung Biomedical Research Institute.

### Chemical reagents and antibodies

AZD1775 was provided by AstraZeneca and dissolved in dimethyl sulfoxide (DMSO). Antibodies against Wee1, p-cdc2 (Tyr15), CDK1, PARP, p-Akt (Ser473), Akt, p-ERK1/2 (Thr202/Thy204), ERK1/2, γH2AX (Ser139), and H2AX were purchased from Cell Signaling Technology. Anti-β-actin was purchased from Santa Cruz Biotechnology.

### Cell viability assay

Cells were seeded in a 96-well plate, allowed to adhere overnight, and treated with the indicated concentrations of AZD1775 for 72 h. Cell viability was determined using a Cell Counting Kit (Dojindo Molecular Technologies) according to the manufacturer's instructions. The subsequent absorbance was measured on an ELISA reader at a wavelength of 450 nm. Long-term viability was assessed by colony formation assay. In brief, cells were seeded in 6-well plates. Following 14 days of treatment, cells were fixed and stained with crystal violet. Colonies with >50 cells were quantified.

### BrdU cell proliferation assay

Cell proliferation was determined using a BrdU Cell Proliferation Assay Kit (Cell Signaling Technology). In brief, cells were seeded in 96-well plates. Following 48 h of treatment, cells were incubated with BrdU for 4 h according to the manufacturer's instructions. After fixation, BrdU (Bu20a) mouse mAb was used to detect BrdU incorporation into DNA.

### Flow cytometric analysis

Cells were treated with AZD1775 (0.5 μM) for 24 h and harvested. After washing with ice-cold PBS, cells were fixed in 70% ethanol at 4°C. Fixed cells were stained with 10 μg/ml RNase A and 20 μg/ml propidium iodide (PI). DNA content was analyzed by flow cytometry (BD Biosciences). Cell cycle was analyzed by gating, and apoptosis was determined using the sub G1 fraction.

### Western blot analysis

Cells and tumor samples were lysed on ice in NP-40 lysis buffer supplemented with a protease and phosphatase inhibitor cocktail (Sigma). Equal amounts of protein were then subjected to SDS-PAGE (NuPAGE 4-12% Bis-Tris Gel; Invitrogen) and transferred to polyvinylidene difluoride (PVDF) membranes. Membranes were incubated with the indicated antibodies and developed by ECL.

### Immunocytochemistry and immunohistochemistry

For immunocytochemistry, cells were cultured on coverslips and treated with AZD1775 (0.5 μM) for 24 h. After fixation with 3.7% formaldehyde, cells were permeabilized with 0.5% Triton X-100 and blocked with 5% BSA. Then, the cells were incubated with γH2AX (1:200) antibody and conjugated with secondary antibodies labeled with Alexa Fluor 594. Cell nuclei were counterstained with 4’,6-diaidino-2-phenylindole (DAPI), and slides were mounted with coverslips for analysis with a fluorescence microscope (OLYMPUS). Immunohistochemical staining was performed on formalin-fixed, paraffin-embedded tissues from mice xenografts, sectioned at a 5-μm thickness.

### *In vivo* xenograft studies

All procedures involving animals were reviewed and approved by the Institutional Animal Care and Use Committee (IACUC) at Samsung Biomedical Research Institute (SBRI). SBRI is an Association of Assessment and Accreditation of Laboratory Animal Care International (AAALAC International) accredited facility and abides by the Institute of Laboratory Animal Resources (ILAR) guidelines. Six-week-old BALB/c female nude mice were injected subcutaneously with either A549 (5×10^6^) or Calu-6 (5 × 10^6^) cells. When tumor sizes reached approximately 100 mm^3^, mice were randomized by tumor size and subjected to the assigned treatment. At least five mice per treatment group were included. AZD1775 was dissolved in 0.5% methylcellulose. Each group of mice was dosed via oral gavage with vehicle (once daily) or AZD1775 (30 mg/kg/d; once daily or 60 mg/kg/d; twice daily). Tumor size and body weight were measured twice weekly. Tumor volumes were calculated using the following formula: V = (L x W^2^)/2 (L: length; W: width). The tumors were removed for Western blot analysis and immunohistochemistry.

### Statistical analysis

Data are presented as the mean ± SEM. Statistical analyses were conducted using GraphPad Prism (GraphPad software). Data were analyzed using two-tailed unpaired Student's *t*-test for comparisons of two groups. *P*-values <0.05 were considered statistically significant.

## Abbreviations

NSCLC: non-small cell lung cancer
HNSCC: head and neck squamous cell carcinoma
*KRAS*^MUT^: *KRAS*-mutant
*KRAS*^WT^: *KRAS* wild-type
*TP53*^MUT^: *TP53*-mutant
*TP53*^WT^: *TP53* wild-type
*STK11*^MUT^: *STK11*-mutant
PI: propidium iodide

## References

[1] Karachaliou N, Mayo C, Costa C, Magri I, Gimenez-Capitan A, Molina-Vila MA, Rosell R (2013). KRAS mutations in lung cancer. Clinical lung cancer.

[2] Lee YS, Bae SC (2016). How do K-RAS-activated cells evade cellular defense mechanisms?. Oncogene.

[3] Richer AL, Friel JM, Carson VM, Inge LJ, Whitsett TG (2015). Genomic profiling toward precision medicine in non-small cell lung cancer: getting beyond EGFR. Pharmacogenomics and personalized medicine.

[4] Grabocka E, Pylayeva-Gupta Y, Jones MJ, Lubkov V, Yemanaberhan E, Taylor L, Jeng HH, Bar-Sagi D (2014). Wild-type H- and N-Ras promote mutant K-Ras-driven tumorigenesis by modulating the DNA damage response. Cancer cell.

[5] Syljuasen RG, Hasvold G, Hauge S, Helland A (2015). Targeting lung cancer through inhibition of checkpoint kinases. Frontiers in genetics.

[6] Grabocka E, Commisso C, Bar-Sagi D (2015). Molecular pathways: targeting the dependence of mutant RAS cancers on the DNA damage response. Clinical cancer research.

[7] Weisberg E, Nonami A, Chen Z, Liu F, Zhang J, Sattler M, Nelson E, Cowens K, Christie AL, Mitsiades C, Wong KK, Liu Q, Gray N, Griffin JD (2015). Identification of Wee1 as a novel therapeutic target for mutant RAS-driven acute leukemia and other malignancies. Leukemia.

[8] Matheson CJ, Backos DS, Reigan P (2016). Targeting WEE1 Kinase in Cancer. Trends in pharmacological sciences.

[9] Harper JW, Elledge SJ (2007). The DNA damage response: ten years after. Molecular cell.

[10] Vriend LE, De Witt Hamer PC, Van Noorden CJ, Wurdinger T (2013). WEE1 inhibition and genomic instability in cancer. Biochimica et biophysica acta.

[11] De Witt Hamer PC, Mir SE, Noske D, Van Noorden CJ, Wurdinger T (2011). WEE1 kinase targeting combined with DNA-damaging cancer therapy catalyzes mitotic catastrophe. Clinical cancer research.

[12] Lal S, Zarei M, Chand SN, Dylgjeri E, Mambelli-Lisboa NC, Pishvaian MJ, Yeo CJ, Winter JM, Brody JR (2016). WEE1 inhibition in pancreatic cancer cells is dependent on DNA repair status in a context dependent manner. Scientific reports.

[13] Aarts M, Sharpe R, Garcia-Murillas I, Gevensleben H, Hurd MS, Shumway SD, Toniatti C, Ashworth A, Turner NC (2012). Forced mitotic entry of S-phase cells as a therapeutic strategy induced by inhibition of WEE1. Cancer discovery.

[14] Magnussen GI, Hellesylt E, Nesland JM, Trope CG, Florenes VA, Holm R (2013). High expression of wee1 is associated with malignancy in vulvar squamous cell carcinoma patients. BMC cancer.

[15] Music D, Dahlrot RH, Hermansen SK, Hjelmborg J, de Stricker K, Hansen S, Kristensen BW (2016). Expression and prognostic value of the WEE1 kinase in gliomas. Journal of neuro-oncology.

[16] Iorns E, Lord CJ, Grigoriadis A, McDonald S, Fenwick K, Mackay A, Mein CA, Natrajan R, Savage K, Tamber N, Reis-Filho JS, Turner NC, Ashworth A (2009). Integrated functional, gene expression and genomic analysis for the identification of cancer targets. PloS one.

[17] Yoshida T, Tanaka S, Mogi A, Shitara Y, Kuwano H (2004). The clinical significance of Cyclin B1 and Wee1 expression in non-small-cell lung cancer. Annals of oncology.

[18] Rajeshkumar NV, De Oliveira E, Ottenhof N, Watters J, Brooks D, Demuth T, Shumway SD, Mizuarai S, Hirai H, Maitra A, Hidalgo M (2011). MK-1775, a potent Wee1 inhibitor, synergizes with gemcitabine to achieve tumor regressions, selectively in p53-deficient pancreatic cancer xenografts. Clinical cancer research.

[19] Kreahling JM, Gemmer JY, Reed D, Letson D, Bui M, Altiok S (2012). MK1775, a selective Wee1 inhibitor, shows single-agent antitumor activity against sarcoma cells. Molecular cancer therapeutics.

[20] Bridges KA, Hirai H, Buser CA, Brooks C, Liu H, Buchholz TA, Molkentine JM, Mason KA, Meyn RE (2011). MK-1775, a novel Wee1 kinase inhibitor, radiosensitizes p53-defective human tumor cells. Clinical cancer research.

[21] Leijen S, van Geel RM, Sonke GS, de Jong D, Rosenberg EH, Marchetti S, Pluim D, van Werkhoven E, Rose S, Lee MA, Freshwater T, Beijnen JH, Schellens JH (2016). Phase II Study of WEE1 Inhibitor AZD1775 Plus Carboplatin in Patients With TP53-Mutated Ovarian Cancer Refractory or Resistant to First-Line Therapy Within 3 Months. J Clin Oncol.

[22] Guertin AD, Li J, Liu Y, Hurd MS, Schuller AG, Long B, Hirsch HA, Feldman I, Benita Y, Toniatti C, Zawel L, Fawell SE, Gilliland DG, Shumway SD (2013). Preclinical evaluation of the WEE1 inhibitor MK-1775 as single-agent anticancer therapy. Molecular cancer therapeutics.

[23] Van Linden AA, Baturin D, Ford JB, Fosmire SP, Gardner L, Korch C, Reigan P, Porter CC (2013). Inhibition of Wee1 sensitizes cancer cells to antimetabolite chemotherapeutics in vitro and in vivo, independent of p53 functionality. Molecular cancer therapeutics.

[24] Do K, Wilsker D, Ji J, Zlott J, Freshwater T, Kinders RJ, Collins J, Chen AP, Doroshow JH, Kummar S (2015). Phase I Study of Single-Agent AZD1775 (MK-1775), a Wee1 Kinase Inhibitor, in Patients With Refractory Solid Tumors. J Clin Oncol.

[25] Kao M, Green C, Sidorova J, Mendez E (2017). Strategies for Targeted Therapy in Head and Neck Squamous Cell Carcinoma Using WEE1 Inhibitor AZD1775. JAMA Otolaryngol Head Neck Surg.

[26] Lescarbeau RS, Lei L, Bakken KK, Sims PA, Sarkaria JN, Canoll P, White FM (2016). Quantitative Phosphoproteomics Reveals Wee1 Kinase as a Therapeutic Target in a Model of Proneural Glioblastoma. Molecular cancer therapeutics.

[27] Pfister SX, Markkanen E, Jiang Y, Sarkar S, Woodcock M, Orlando G, Mavrommati I, Pai CC, Zalmas LP, Drobnitzky N, Dianov GL, Verrill C, Macaulay VM (2015). Inhibiting WEE1 Selectively Kills Histone H3K36me3-Deficient Cancers by dNTP Starvation. Cancer cell.

[28] Dietlein F, Kalb B, Jokic M, Noll EM, Strong A, Tharun L, Ozretic L, Kunstlinger H, Kambartel K, Randerath WJ, Jungst C, Schmitt A, Torgovnick A (2015). A Synergistic Interaction between Chk1- and MK2 Inhibitors in KRAS-Mutant Cancer. Cell.

[29] Skoulidis F, Byers LA, Diao L, Papadimitrakopoulou VA, Tong P, Izzo J, Behrens C, Kadara H, Parra ER, Canales JR, Zhang J, Giri U, Gudikote J (2015). Co-occurring genomic alterations define major subsets of KRAS-mutant lung adenocarcinoma with distinct biology, immune profiles, and therapeutic vulnerabilities. Cancer discovery.

[30] Chen Z, Cheng K, Walton Z, Wang Y, Ebi H, Shimamura T, Liu Y, Tupper T, Ouyang J, Li J, Gao P, Woo MS, Xu C (2012). A murine lung cancer co-clinical trial identifies genetic modifiers of therapeutic response. Nature.

[31] Do K, Doroshow JH, Kummar S (2013). Wee1 kinase as a target for cancer therapy. Cell cycle.

[32] Dominguez-Kelly R, Martin Y, Koundrioukoff S, Tanenbaum ME, Smits VA, Medema RH, Debatisse M, Freire R (2011). Wee1 controls genomic stability during replication by regulating the Mus81-Eme1 endonuclease. The Journal of cell biology.

[33] Cuneo KC, Morgan MA, Davis MA, Parcels LA, Parcels J, Karnak D, Ryan C, Liu N, Maybaum J, Lawrence TS (2016). Wee1 Kinase Inhibitor AZD1775 Radiosensitizes Hepatocellular Carcinoma Regardless of TP53 Mutational Status Through Induction of Replication Stress. International journal of radiation oncology, biology, physics.

[34] Murrow LM, Garimella SV, Jones TL, Caplen NJ, Lipkowitz S (2010). Identification of WEE1 as a potential molecular target in cancer cells by RNAi screening of the human tyrosine kinome. Breast cancer research and treatment.

[35] Beck H, Nahse V, Larsen MS, Groth P, Clancy T, Lees M, Jorgensen M, Helleday T, Syljuasen RG, Sorensen CS (2010). Regulators of cyclin-dependent kinases are crucial for maintaining genome integrity in S phase. The Journal of cell biology.

[36] Beck H, Nahse-Kumpf V, Larsen MS, O'Hanlon KA, Patzke S, Holmberg C, Mejlvang J, Groth A, Nielsen O, Syljuasen RG, Sorensen CS (2012). Cyclin-dependent kinase suppression by WEE1 kinase protects the genome through control of replication initiation and nucleotide consumption. Molecular and cellular biology.

